# A mixed-methods assessment of understanding (AoU) tool for AIDS vaccine trials in sub-Saharan Africa: results from a pilot study

**DOI:** 10.1186/1742-4690-9-S2-P135

**Published:** 2012-09-13

**Authors:** G Lindegger, M Quayle, S Singh, S Welsh, D Mark, M Wallace, S Roux, L Bekker, L Mwananyanda, W Kilembe, E Chomba, S Allen, F Priddy, P Fast

**Affiliations:** 1 School of Psychology, University of Kwa-Zulu Natal, Pietermaritzburg, South Africa; 2 GHAR Consulting Inc, New York, NY, USA; 3 Desmond Tutu HIV Foundation, University of Cape Town, Cape Town, South Africa; 4 Zambia-Emory HIV Research Project, Lusaka, Zambia; 5 Zambia-Emory HIV Reseach Project, Lusaka, Zambia; 6 Emory University, Atlanta, GA, USA; 7 International AIDS Vaccine Initiative, New York, NY, USA

## Background

Assessments of understanding (AoUs) in clinical trials are often composed of true/false multiple choice questions, however, these tools can be difficult for volunteers with limited education or without prior testing experience.

## Methods

35 adults were recruited at two research centers in Southern Africa. A within-subjects, repeated measures design was used, whereby each volunteer served as his /her own control. An AoU tool with closed- and open-ended questions was administered within a hypothetical AIDS vaccine trial setting. Performance on closed- and open-ended questions was compared using correlations and repeated-measure t-tests, limited to 4 complex concepts: false sense of security, risk of false positive test, need for contraception, and potentially enhanced susceptibility.

## Results

Mean scores of understanding for each concept assessed by closed-ended questions ranged from 0.73 (need for contraception) to 0.84 (risk of false positive test); and by open-ended questions from 0.4 (risk of false positive test) – 0.6 (need for contraception). Scores for the open-ended measure were all lower than the equivalent closed-ended measure. Correlations between the closed- and open-ended measures were generally low, achieving significance for false sense of security (r=0.377), potentially enhanced susceptibility (r=0.393), and total score across concepts (r=0.617). Volunteers’ understanding as assessed by the closed- and open-ended methods differed significantly: false sense of security= -3.862; risk of false positive test= -7.210; need for contraception= -2.303; and potentially enhanced susceptibility= -8.007. The correlation with years of education was consistently and significantly higher for the open-ended measure than the true/false questionnaire with the exception of need for contraception.

## Conclusion

The results suggest the qualitative measure better assesses understanding than the quantitative measure. The scores from the two assessment methods have limited interchangeability. The standard closed-ended questions appear to provide an inflated measure of volunteers’ understanding. An assessment tool with closed- and open-ended questions is better suited to determine genuine understanding.

